# A Qualitative Study of U.S. Latino Fathers’ Perceptions of Parenting Motivations

**DOI:** 10.1007/s10826-025-03238-1

**Published:** 2025-12-08

**Authors:** Rachel A. Ghosh, Natasha J. Cabrera, Yu Chen, Avery Hennigar, Minxuan He, Stephanie M. Reich, Kevin Roy

**Affiliations:** 1 Department of Human Development and Quantitative Methodology, University of Maryland, College Park, MD USA; 2 Department of Pediatrics, Grossman School of Medicine, New York University, New York, NY USA; 3 Mathematica, Washington, D.C USA; 4 Department of Psychology, Mount St. Mary’s University, Emmitsburg, MD USA; 5 School of Education, University of California, Irvine, CA USA; 6 Department of Family Science, University of Maryland, College Park, MD USA

**Keywords:** Motivation, Fathers, Latino, Parenting, Thematic analysis

## Abstract

Though ample research and theory suggest that parents’ beliefs and cognitions are important predictors of their parenting behaviors, there is little understanding of Latino fathers’ perceived parenting motivations. We explored resident, first-time fathers’ motivations to be involved parents in a sample (*N* = 85) of socioeconomically diverse Latino fathers participating in a parenting intervention in the Washington D.C. area and southern California. Data were collected through structured interviews that were recorded during home visits when infants were 18-months old. Bilingual research assistants transcribed and translated into English fathers’ responses to the interview question, “What makes you want to be a good parent?” A thematic analysis revealed five main emergent themes: (1) personal rearing history, (2) desire to rear a well-adjusted child, (3) relationship with their child, (4) intrinsic motivations, and (5) sense of duty and responsibility. We further explored whether fathers’ perceived parenting motivations varied by their nativity status (i.e., U.S.-born or immigrant). We found variations in each of the themes, including that immigrant Latino fathers were more likely to prioritize their children’s morals and values, whereas U.S.-born Latino fathers emphasized their child’s future success. This study contributes to the limited research on Latino fathers’ parenting perceptions and beliefs. The findings can be used to inform programs geared at strengthening Latine family functioning in the face of adversity through leveraging the reasons behind why fathers want to be positively involved with their young children.

## Highlights

This study used thematic analysis to explore the perceived parenting motivations of 85 first-time Latino fathers living in the U.S.

We also explored variation in fathers’ parenting motivations by their nativity status (i.e., U.S.-born vs. immigrant).

We identified five main themes, and various subthemes, in fathers’ perceived motivations, as well as differences by nativity status.

The findings may inform parenting interventions, given the influence of fathers’ motivations on parenting behaviors.

Although everyone has different ideas of what it means to be a “good” parent,” most parents have a general perception, a system of culturally-shaped beliefs and values, of what it means to be a good parent and why they want to be one. For instance, being a “good” or involved parent in the United States generally includes providing for children emotionally and financially, and keeping them safe, sheltered, and healthy (National Academies of Sciences, Engineering, and Medicine, 2016). It also entails engaging in goal-directed behavior, such as spending time, energy, and resources on children, which is driven in part by parents’ parenting motivations. Motivation to act toward a goal (e.g., to be a positively involved parent) is understood as a force that explains why people initiate, sustain, and terminate a certain behavior (Graham & Weiner, 2012). Theoretically and empirically, fathers with strong parenting motivations are more involved with their children and have children with better developmental outcomes (Hofferth et al., 2013). Because parenting motivations are key determinants of parenting behaviors and practices (Bornstein et al., 2018), it is important to understand what motivates men to be “good” and involved parents. Yet, little is known about these cognitions and beliefs early in children’s lives - an important time for growing and learning (Cabrera et al., 2008). We have even less information about perceived parenting motivations among U.S. Latino fathers, who often face myriad socioeconomic challenges that may make parenting more difficult (Cabrera & Bradley, 2012; Cabrera & Hennigar, 2019). In this study, we use thematic analysis to examine the perceived parenting motivations of first-time Latino fathers in the U.S., and we explore differences in their motivations by nativity status (i.e., U.S.-born compared to immigrant).

## Theoretical Framework

We draw upon Cabrera and colleagues’ (2014) expanded heuristic model of father-child relationships that lays out the dynamic and reciprocal processes through which fathers impact their children’s development, as well as fathers’ personal characteristics (e.g., their beliefs, role identity) that predict to parenting behaviors. In particular, this model highlights five potential influences that may shape a father’s parenting beliefs and motivations: (1) his personal history, including his cultural (e.g., race/ethnicity) and rearing history; (2) his social networks and community; (3) household socioeconomic status; (4) other family members’ personalities, characteristics, and behaviors, such as those of the mother and child; and (5) his own parenting behaviors. In addition, there are several indirect influences to fathers’ parenting beliefs, such as his employment situation, his family relationships (e.g., the mother-father or father-child relationship), and his child’s development. Finally, all of these factors are overarchingly influenced by the larger social, political, cultural, and economic conditions in which families are living. We use Cabrera and colleagues’ (2014) model because it explains how individual, child, family, social, and cultural influences play a role in shaping fathers’ cognitive processes and beliefs. The qualitative nature of our study allows for generation of novel insights that can be used to build upon and expand this model to more specifically capture the experiences of Latino fathers.

## Potential Barriers to Parental Involvement for Latino Fathers

Being a highly involved and positively engaged parent is not always easy, especially for new parents who face economic, material, and sociocultural hardships and stressors, such as many first-time U.S. Latino fathers (Lee et al., 2023). Though most U.S. Latino fathers live together with their children (i.e., are resident fathers), this does not necessarily equate with high quality and frequency of father engagement (Karberg et al., 2017). Some studies with national samples of U.S. Latino fathers and their young children find Latino fathers to be positively involved (e.g., spend time with them, show warmth and responsivity) in their children’s lives (Cabrera et al., 2008, 2011; Wildsmith et al., 2020). Other studies using national data and comparing levels of involvement across racial/ethnic groups have found resident Latino fathers of children ages 0 to 18 are less engaged in daily caretaking (e.g., changing diapers, feeding; Jones & Mosher, 2013) and cognitively stimulating activities (e.g., reading books) than are resident Black and White fathers (Wildsmith et al., 2020).

Barriers to parental engagement for Latino fathers could come from individual (e.g., mental health), employment, and cultural sources. For instance, a potential barrier to frequency of involvement might be Latino fathers’ high rates of labor force participation (Karberg et al., 2017). Most Latino fathers, especially immigrant Latinos, report working long hours (Wildsmith et al., 2020). A potential culturally-related barrier could be the circumstances of their migration to America. Latino fathers can face immigration-related separations from their family, or experience racial discrimination, which can result in depression and negatively impact the quantity and quality of involvement (Cabrera & Bradley, 2012; Paredes & Parchment, 2021). Paternal involvement is also related to fathers’ adherence to traditional cultural gender norms such as *machismo* (Planalp et al., 2021) and parenting beliefs that emphasize the mother as the nurturing caretaker and the father’s role as more instrumental (Karberg et al., 2017; Wildsmith et al., 2020). These traditional beliefs may be especially relevant for foreign-born and/or less acculturated Latino fathers. For instance, Cabrera and colleagues (2006) observed that less acculturated U.S. Latino fathers (measured through English language proficiency as a rough proxy) spent less time engaged in caregiving activities and physical play with their infants than did more acculturated U.S. Latino fathers. Ultimately, U.S. Latino fathers often face a set of barriers that may negatively impact their level of father-child engagement, which may in turn compromise their children’s developmental outcomes. Fathers’ sense of motivation may be a key factor in maintaining high levels of engagement and positive interactions with children (Wildsmith et al., 2020), despite socioeconomic barriers and contextual difficulties.

## What Motivates Latino Fathers to be Engaged Parents?

Because research on parenting motivations among Latino fathers is limited, we review the few studies that identify fathers’ (and mothers’) motivations to parent more broadly, and we review studies on Latine parents whenever possible. We organize this literature review in line with contextual influences described in Cabrera and colleagues’ (2014) theoretical model that are relevant to parenting motivations, namely: fathers’ rearing history, personal characteristics, the family context, and the cultural context.

### Fathers’ Rearing History

According to Cabrera and colleagues (2014), fathers’ rearing history and childhood experiences with their own parents are predictive of their personal characteristics (e.g., their parenting motivations). A study of 185 African American men with adolescent children showed that men’s intergenerational factors (e.g., childhood involvement with their father, beliefs about their father figure) were related to their fathering role ideologies (Cooper et al., 2019). Oftentimes, the effect of men’s experiences with their parents on their own beliefs and parenting is framed through either a compensation hypothesis or a modeling hypothesis (Floyd & Morman, 2000). That is, fathers either report having a negative childhood and parenting experience that motivated them to compensate with their own children, or they had a positive experience that they wanted to model after. A similar idea was expressed among a sample of Mexican and U.S. Latino fathers, in that the men described both a transmission of (i.e., parenting similarly) and a transformation of the fathering values and behaviors they had experienced with their own fathers (Taylor & Behnke, 2005).

### Fathers’ Personal Characteristics

Fathers may also be driven to be involved parents by their personal characteristics, such as their goals and aspirations, their sense of parenting competence, and their fatherhood identity (i.e., their beliefs, attitudes, and internalized perceptions of the father role; de Haan et al., 2009; Hofferth et al., 2013; Macon et al., 2017; Mogro-Wilson & Cifuentes, 2020). A study with low-income, racially diverse fathers found that fathers who perceived their parenting role as an investment in their children’s development had more caregiving involvement than those who perceived financial provision as their primary parenting role (Macon et al., 2017). Alternatively, a study of expectant Canadian fathers found that men with stronger biological essentialist beliefs (e.g., believing women are better caregivers than men) had weaker intentions regarding their future childcare involvement, whereas men with a greater sense of control over external barriers in their life had stronger involvement intentions (Ross-Plourde et al., 2022). Mogro-Wilson and colleagues (2020) found in a sample of 309 Latino fathers that those who strongly identified as fathers (i.e., had a higher salience of the father role) reported less problem drinking. Having a strong identity as fathers also acted as a protective mechanism against the negative effects of machismo on problem drinking. Moreover, fathers’ sense of interest, satisfaction, challenge, or meaningfulness regarding the fathering role and identity, or their feelings of internal and external pressures to be involved, are also motivating factors (Jungert et al., 2015). In a qualitative study of immigrant Latine mothers (*n* = 20) and fathers (*n* = 10) with young children (ages two to four), parents’ personal experiences with external adversities were a main source of motivation to do better for themselves and their children (Aldoney & Cabrera, 2016). In an explorative study of five Latino fathers, the men viewed themselves as personally responsible to be involved in their child’s education, and drew upon their own experiences with schooling, as well as their religious beliefs, as motivating factors (FitzGerald et al., 2019).

### Family Context

Cabrera and colleagues’ (2014) model describes several influential aspects of the family context, including child characteristics, the father-child relationship, and the mother-father relationship. Fathers may feel motivated as parents because they love their children and want to ensure their children’s wellbeing (Li & Meier, 2017). Aldoney and Cabrera (2016) found that immigrant Latine parents with young children wanted to make sacrifices for their children’s wellbeing and education, and Latino fathers deeply believed in the importance of children and their innate responsibility to care for and promote the success of their children. Additionally, FitzGerald and colleagues (2019) found that fathers felt motivated to support their children’s educational success in part because of invitations they received from their children to be involved in their schooling. Relatedly, Taylor and Behnke (2005) found that Latino fathers were concerned with generativity and intergenerational transmission of values to their children, especially the importance of a good education, strong work ethic, and *respeto* (i.e., respecting parents and elders; Cruz et al., 2011). At a family level, fathers may be motivated by their partner or spouse, such as through their perceptions of coparenting support (Ross-Plourde et al., 2022). For instance, Bouchard and colleagues (2007) found with a sample of French-Canadian fathers of preschoolers that men’s perceived partner support for their parenting competence was related to fathers’ own sense of parenting competence, which was then related to fathers’ parenting motivations (Bouchard et al., 2007).

### Cultural Context

The cultural context in which fathers live and rear their children shapes their parenting beliefs, including their motivations to be involved parents (Planalp et al., 2021). Cultural norms and values are important determinants of expected and socialized behavior in each cultural group. Latine cultural values such as *respeto*, *familismo*, *machismo*, and *caballerismo* emphasize respect, commitment to family, and masculine gender roles. These values are important to Latino fathers (especially foreign-born and/or less acculturated U.S. Latino fathers) and for shaping fathering behaviors (Cabrera & Bradley, 2012; Mogro-Wilson & Cifuentes, 2021; Planalp et al., 2021). The cultural value of *personalismo* prioritizes strong, loyal, and deeply personal connections, emphasizing trust and warmth over impersonal authority. Though research has rarely explored how Latino fathers express *personalismo*, it has been suggested as a potential motivating force for Latino fathers in developing nurturing and emotionally engaged relationships with their children (Mogro-Wilson & Cifuentes, 2021).

Aldoney and Cabrera (2016) found that immigrant Latine parents highly prioritized socializing their toddlers to have Latine cultural values and beliefs such as *respeto* and a strong sense of family, as well as American cultural values such as autonomy. Further, a study of Mexican-origin U.S. fathers found that fathers with higher levels of “positive *machismo*” (i.e., *caballerismo*, including dignity, honor, respect, and commitment to family and providing; Arciniega et al., 2008) had 5th -grade children who reported greater positive father involvement than fathers with lower positive *machismo* (Cruz et al., 2011). Similarly, Mogro-Wilson and Cifuentes (2021) found that Latino fathers with higher reported levels of *personalismo* and *caballerismo* and lower levels of traditional *machismo* reported using more authoritative parenting practices, which are often associated with the most optimal child developmental outcomes. In a study of low-to-moderate income Mexican-American fathers with 5th -grade children, Mexican-identified men were more likely than U.S.-acculturated men to engage in caretaking and participate in traditionally “feminine” activities, contrary to traditional *machismo* stereotypes (Coltrane et al., 2004). In sum, studies indicate that Latino fathers may feel encouraged to be involved parents because of their adherence to cultural norms and values that emphasize fulfillment of family roles and maintaining close family relationships (Cabrera & Bradley, 2012).

Overall, there is little information about the reasons that motivate Latino fathers to be engaged parents despite the challenges and barriers they may face. The few existing studies with Latino fathers do not shed light on the types of perceived motives that underly their fathering behaviors (Cabrera & Bradley, 2012). Theoretically, multiple factors at different levels of the ecological system encourage parents to be involved, such as their rearing history, love of their child, and cultural values. But, as described by Cabrera and Bradley (2012), “Research on Latino fathers is insufficient to warrant definite or highly specific conclusions…and it is not at all clear how findings apply across Latino subgroups” (pp. 235–236). Accordingly, more research is needed to understand the within-group heterogeneity of Latino fathers’ perceived parenting motivations among a larger and more diverse sample of Latinos with young children.

## Differences in Parenting Motivations by Nativity Status

As emphasized by recent events, immigration is a source of political and social strife in the United States. Regardless of their level of documentation, immigrants, who are born elsewhere, have a different set of lived experiences and access to resources that can have short- and long-term consequences for their family’s well-being (Campos, 2008). Nativity status (in this context, U.S.-born or foreign-born) is an important lens through which to understand variability in Latino fathers’ parenting motivations because it reflects differences in men’s context, culture, and life experiences, which differentially shape one’s beliefs and cognitions (Campos, 2008; Karberg et al., 2017). Efforts to understand the heterogeneity among Latines have revealed significant differences by nativity status in Latino fathers’ characteristics, values, beliefs, and parenting behaviors (Karberg et al., 2017; Planalp et al., 2021; Taylor & Behnke, 2005; Wildsmith et al., 2020). Moreover, immigrant fathers may face a distinct set of pressures and barriers in their day-to-day lives compared to U.S.-born fathers, which can uniquely shape their beliefs and behaviors. For instance, immigrant Latino fathers report significantly lower incomes and education levels than U.S.-born fathers (Karberg et al., 2017; Wildsmith et al., 2020), and they more often work non-standard hours (Crosby & Mendez, 2017), all of which can impact their parenting (Cabrera et al., 2014). Indeed, Wildsmith and colleagues (2020) found that immigrant Latino fathers reported engaging in fewer daily activities (e.g., reading, bathing, playing) with their young children (ages zero to four years) than did U.S.-born Latino fathers.

Additionally, immigrant Latino fathers tend to endorse more traditional views from their home cultures on parenting roles (Karberg et al., 2017; Planalp et al., 2021; Wildsmith et al., 2020). D’Angelo and colleagues (2012) found with a large dataset (*N* = 787) that immigrant Latino fathers showed lower levels of caretaking and positive engagement with their infants than did U.S.-born fathers, which was partially explained through immigrant fathers’ more traditional attitudes (e.g., gender division of labor in the home). Although we did not find any studies that examined nativity status differences in Latino fathers’ parenting motivations, there is ample evidence to suggest that immigrant and U.S.-born Latino fathers have meaningfully different parenting experiences, beliefs, values, and behaviors. Therefore, we would expect Latino fathers’ perceived parenting motivations may also differ based on nativity status, and this topic warrants further exploration.

## The Current Study

There is limited empirical evidence that identifies the sources from which Latino fathers draw meaning to sustain positive engagement with their young children (i.e., being a “good” father). Much of our current understanding of parenting beliefs and cognitions comes from studies with mothers, whereas few studies have examined variation in perceptions of parenting motivations with a sample of ethnic-minority, socioeconomically diverse fathers, particularly those with infants. Thus, we draw on Cabrera and colleagues’ (2014) model of father-child relationships and explore the following question using thematic analysis: How do Latino fathers in the U.S. describe what motivates them to be “good” parents? In order to explore the variability in our sample of 85 first-time fathers with 18-month-old children, we also explore descriptive differences in perceived parenting motivations between U.S.-born and immigrant Latino fathers.

## Method

### Participants

Data for this study were drawn from a subset of the Baby Books 2 (BB2) project (Cabrera & Reich, 2017). BB2 is an NICHD-funded, bilingual (i.e., English, Spanish), eight-wave parenting intervention involving 210 racially/ethnically and economically diverse families from the greater Washington D.C. area and Orange County, California. Heterosexual couples were recruited from community spaces (e.g., community centers, physician offices, hospital waiting rooms, farmers’ markets). Parents had to meet the following criteria to qualify for the initial home visit: be co-residing (i.e., mother, father, and child must be living together), be first-time parents, and have an annual household income of up to $75,000 or 300% of the federal poverty line for a family of three.

For the current study, fathers who self-identified as Latino or Hispanic and had complete data at the 18-month home visit were included (*N* = 85). The fathers in this sample ranged in age from 18 to 41 years (*M* = 28.5, *SD* = 6.0). Their level of education varied: 28% had less than a high school education, 25% had a high school degree or equivalent, and 47% had some college or more (see Table 1 for sample demographics). Most of the fathers in this sample were immigrants and born outside of the United States (*n* = 58; 68%). The majority of fathers were English/Spanish bilingual (73%). There was also variation in reported household income: 24% of fathers reported household incomes of $20,000 or less per year, while another 24% of fathers reported an annual family income of $60,000 or more. All of the fathers lived with their child and their child’s mother; 45% were married and 42% reported “living as married/engaged.”

**Table 1:** Sample Characteristics of 85 Latino Fathers

	*N* (%)	M (SD)	Range
Age		28.5 (5.8)	18–41
Yearly Income		$39,140 ($23,400)	$0-100,000
Education			
Less than High School	24 (28%)		
High School or Equivalent	21 (25%)		
At Least Some College	40 (47%)		
Born in the U.S.	27 (32%)		
Born Outside the U.S.	58 (68%)		
Years Living in the U.S.		12.4 (7.9)	1–37
Born in Mexico	19 (22%)		
Born in El Salvador	15 (18%)		
Born in Guatemala	11 (13%)		
Born in Ecuador	3 (3.5%)		
Born in Honduras	3 (3.5%)		
Born in Peru	3 (3.5%)		
Born in Bolivia	2 (2.4%)		
Born in Venezuela	1 (1.2%)		
Born in Columbia	1 (1.2%)		
Language			
Bilingual English/Spanish	62 (73%)		
Monolingual Spanish	15 (18%)		
Multilingual	6 (7%)		
Marital Status			
Married	38 (45%)		
Living as Married/Engaged	36 (42%)		
Single/Never Married	11 (13%)		
Pregnancy was Planned	53 (62%)		
Site			
Southern California	45 (53%)		
Washington D.C. Area	40 (47%)		
Child Sex: Girl	49 (58%)		

### Data Collection

Data for this study were collected during home visits when children were 18-months-old by a team of Spanish-English bilingual researchers. Researchers used a structured interview procedure and script, and began by saying, “Now, I’d like to ask you some questions about being a parent,” or the equivalent sentence in Spanish if the father identified Spanish as his preferred language (“Ahora le queremos hacer algunas preguntas sobre ser padre”). Then, the researchers asked fathers a set of six open-ended questions, and provided ample time for fathers to give their verbal responses. For the current study, only responses to the following question were analyzed: “What makes you want to be a good parent?”; “¿Qué le hace querer ser un buen padre?” due to our specific interest in fathers’ perceived parenting motivations. The researchers were also instructed: “If parents don’t understand the questions, try rephrasing them or ask them to provide examples,” though this was infrequently required. On average, fathers did not have extensive responses to the set of questions (i.e., interviews lasted around five to eight minutes on average), and fathers’ responses were video-recorded on an iPad. All fathers provided written informed consent for their participation in this study at the time of original recruitment. All procedures and materials for the study were reviewed and approved by the Institutional Review Boards at both institutions affiliated with the study.

### Interview Transcriptions

A team of 15 Spanish-English trained bilingual research assistants (RAs) transcribed the recorded interviews verbatim. Interviews conducted in Spanish were transcribed and then translated into English. After the initial transcription/translation, a second group of RAs read the transcriptions as they watched the videos. Any discrepancies between the video and the transcription/translation document were corrected. A third RA verified the transcript by also watching the interview video and reading the transcript.

### Thematic Analysis

We used a six-phase thematic analysis to identify, analyze, and interpret themes in our data (Braun & Clarke, 2006, 2012; Terry et al., 2017), by becoming familiar with the data, generating initial codes, searching for themes, reviewing themes, defining and naming themes, and producing the report. This process was done using an inductive or “bottom-up” approach, meaning our themes were data-driven and strongly tied to the data themselves (Braun & Clarke, 2006). However, as developmental scientists who are familiar with the fatherhood literature, we inevitably applied our own positioning and theoretical lens (Terry et al., 2017) which can narrow our “analytic field of vision” (Braun & Clarke, 2006, p. 86).

Each member of our research team began by reading the transcripts several times to gain familiarity with the data. We then took preliminary notes and made memos about our global impressions and thought processes, which was followed by initial code generation to identify recurrent patterns. We structured these as potential codes with labels, descriptions, and example quotes and excerpts from the interviews in a shared spreadsheet. We also added our thoughts, questions, ideas, and memos on the spreadsheet so everyone could view each other’s memos. We allowed the data to invoke and provoke a set of meaningful labels, and we named the various extracts of data to define our emergent codes (e.g., “intergenerational,” “child happiness”). Most of this semantic coding (i.e., identifying themes at a ‘surface’ level based on what participants explicitly said; Braun & Clarke, 2006) was first done independently. We then met once a week as a team over Zoom during a period of six months to discuss our thoughts and to reach consensus on our set of codes. We engaged in lengthy dialogue and debate on our choice of language and interpretations of meaning, with all changes to the codebook requiring agreement from all four researchers doing the thematic analysis. We also brought with us sensitizing concepts to the analysis (i.e., existing theoretical ideas and concepts that helped guide our inquiry).

Next, we progressed to searching for, reviewing, and defining themes by consolidating, expanding, comparing, and modifying our codes and themes. For example, our theme “intergenerational motivations” was divided into several distinct subthemes or dimensions of the theme (e.g., “wants to be like his parents,” “wants to be different than his parents,” “didn’t have a father”). This step involved group discussion and consensus building among the researchers, as we re-assembled the fragmented data by examining how different codes and themes fit together. We worked on generating specific definitions and names for each main theme and dimensions of the themes (i.e., subthemes), and we examined the overall story being communicated through our data (Braun & Clarke, 2006). This ended with a final codebook. However, after taking a three-month pause from the project, we completed another round of transcript reading and engaged in another, briefer round of reviewing and defining our themes. This resulted in the emergence of several codes we had not clearly observed before (e.g., “wanting his child to have more than what he had”), removal of codes that were deemed less salient, and some re-organization and re-definition of our existent codes and categories (e.g., “relationship with his child” became a main theme, rather than a dimension under the previous main theme of “motivated by sense of family”). Finally, three researchers from the team (including the first author) used Dedoose software to code the 85 transcripts using the established codebook. Each transcript was coded by at least two of the researchers in order to establish consensus, and the codebook continued to be updated throughout this coding process whenever necessary.

### Data Quality

To establish a rigorous and credible process, we used several commonly recommended strategies based on Guba’s (1981) model of trustworthiness of qualitative research, including reflexivity (i.e., assessing the influence of our own personal history, background, perceptions, and interests on the research process), triangulation of investigators (a team of five researchers), and peer examination/peer debriefing with multiple impartial colleagues versed in qualitative methods and fatherhood research. Regarding reflexivity, as a team of all women, one of our primary barriers was our lack of matching identity with the father participants. Further, as primarily non-Latine researchers, we brought to the data analysis a particular set of norms, values, beliefs, and expectations. The identity differences between us as researchers and the fathers in this study likely influenced our interpretations of their interview responses, leading to more etic than emic interpretations. However, given that this study relies on structured and fairly brief interviews with fathers, the analysis did not require extensive personal interpretation.

To aid in determining the dependability of the findings (i.e., the consistency), we provide a rich description of our research methods in this paper (e.g., data gathering, analysis, and interpretation), and we engaged in a code-recode procedure for peer examination. We chose not to calculate statistical measures of inter-coder reliability because some qualitative researchers have argued this measure to be unwarranted or inappropriate (O’Connor & Joffe, 2020). Further, researchers have argued that this type of numerical measure may be less important than peer debriefing and reaching consensus as a team.

### Differences by Nativity Status

We further investigated patterns and frequencies among the coded excerpts to identify descriptive differences in the themes between immigrant versus U.S.-born fathers. We used the “Code Application by Descriptor” charts in Dedoose (i.e., “Codes x Descriptor”) with the normalize function turned on (i.e., to normalize the raw counts based on the demographic descriptor ratio; Dedoose, n.d.). These charts represent the number of excerpts that have been associated with a particular code separately by each sub-group within the descriptor field (e.g., U.S.-born versus immigrant groups within the nativity status descriptor). We used the normalize function in Dedoose because a graphical representation for code application frequency by sub-group can be misleading if there are unequal numbers of individual cases (i.e., fathers) across the sub-groups (Dedoose, n.d.). Specifically, in our sample, our group of immigrant fathers is disproportionally larger (68%) than the group of U.S.-born fathers (32%). Regarding each proportion we report below, the appropriate interpretation would be: “Of the fathers who were coded as having X motivation (e.g., “motivated by their rearing history”), Y% were immigrant (or U.S.-born) fathers.” Notably, this was not a formal statistical test of differences in fathers’ motivations by nativity status, but rather a qualitative investigation of differences in our applied codes based on graphs of the relative frequencies.

## Findings

Five themes emerged from the data regarding how first-time Latino fathers described their perceptions of what made them want to be positively involved parents: (1) personal rearing history, (2) desire to rear a well-adjusted child, (3) relationship with their child, (4) intrinsic motivations, and (5) sense of duty and responsibility. We also examined the themes that were most salient for immigrant versus U.S.-born fathers in order to more deeply explore the variability in our sample.

### Motivated by their Personal Rearing History

When asked what makes them want to be “good” parents, fathers frequently discussed their own experiences, positive or negative, of being parented and how it motivated them with their children. Four dimensions of this theme emerged: (a) modeling their own practices after their parents/childhood experiences, (b) rejecting/avoiding parenting as they were parented, (c) wanting their child to have more than what they had growing up, and (d) lacking a father role model growing up. Many fathers, the majority of whom were immigrant (66%), shared positive experiences growing up and described wanting to model their parenting based on their own parents, reflecting the cultural value of *respeto*. They described re-living their positive childhood experiences with their own child. For instance, an immigrant Peruvian father described how he was inspired by his own father:

What drives me to be a good parent is looking at the success that my father has created with us as a family. I think we all came out pretty okay in terms of our siblings and looking back at it…I guess I can say it’s a tribute to the parenting that our parents gave us…to be able to just come out in the real world and make good choices because there is always…the right path and the wrong path in everything we do.

Similarly, a U.S.-born Latino father stated he, too, was motivated by his father, “I don’t know, he [child] just reminds me of myself when I was younger and I know how much my dad and them cared for me so it’s, you know, lead by example…” Other fathers shared how they rejected and avoided the way their own parents reared them. This was more common among immigrant fathers (60%), who intentionally strived to be different from their parents. For instance, a U.S.-born Mexican-American father said:

Oh, the fact that my relationship with my father was not perfect growing up, and not because of me but because of issues, my parent’s marriage. And I feel that I need to give my son the best blueprint that I can give him.

Moreover, a Latino father born in El Salvador reported that he wanted to break away from traditional Hispanic cultural norms and opposed the traditional use of corporal punishment among Latine parents. He hoped to break this cycle of violence and educate his child through different means:

What motivates me to be a good dad is not repeating the chain that one brings like the family. Because in the Hispanic culture…we always say ‘I raise my kids because this is how they raised me’ so, but that is like a taboo that us Hispanics have especially in the Hispanic culture, but I do not want, I do not want to raise my daughter like they raised me because a lot of things occurred…hitting. Like one who is Hispanic says you educate with hitting. I personally do not think that with hitting you educate a child, so not committing the same mistakes that were made with me, that is a priority.

Some fathers, especially those born in the U.S. (over 3/4), wanted their child to “have more” or have a better life than what they themselves had growing up. These fathers did not criticize their own parents, they just wanted different for their children. For example, a father born in Honduras said “Just to give my child, it sounds cliché, but, a better life than I had.” Furthermore, although some fathers, only U.S.-born, had no father role model growing up, they wanted to be a good role model to their child. A U.S.-born Latino father said:

I was raised without a dad so I want her to have what I did not have and I just want to see my kid be successful in life, like you know, do things that I didn’t do that I wish I would have done but couldn’t have.

Another U.S.-born father of Mexican and Salvadorian origin said that he did not grow up with a father, and he would not want to put his son through that experience:

I grew up pretty much without a father figure, so I always told myself that I wanted more for my child. I wouldn’t want him to, I wouldn’t want him or her to go through what I went through growing up.

### Motivated by their Desire to Rear a Well-Adjusted Child

Fathers were also determined to be engaged parents so that their children would develop into well-adjusted individuals. This theme included five dimensions focused on fathers’ goals for their child: (a) happiness/healthiness, (b) development of good morals and values, (c) becoming a good person/member of society, (d) having the best/having everything/having resources, and (e) successful future and life. Many fathers described wanting to be a “good” parent simply because they wanted their child to be happy and healthy (e.g., “…making sure everything is fine and that he has very good health.”) and several expressed how that goal was rewarding in itself (e.g., “Just seeing her smile every day.”) Similarly, fathers often reported wanting their child to “have the best” or “have everything,” and some were specific to opportunities, materials, and education. For instance, a Salvadorian-born father stated “Giving him a good clean healthy space, making sure he always has his food, toys to play with, giving him everything he needs, that he has clothes.”

Related to this idea were fathers’ perceived motivations to ensure their child would have a successful life and future. Though individual fathers may define success differently, various men (over half of whom were U.S.-born), made statements such as: “I just want to see my kid…be successful in life” and “So that (she) can be happy, have the best future possible.” These men were focused on the more distant future. Another aspect of child adjustment that motivated fathers, mostly commonly immigrant fathers (75%), was for their children to have good morals and values, related to the cultural value of *educación* (importance of socialization and manners). One father born in Ecuador said, “It is important to be a good father so that we can leave children with good teachings, children with good values, children with good principles, hardworking children, honest children.” As included within these principles, some fathers described wanting their children to value the family unit and uphold family traditions. A Latino father born in Colombia said:

What makes me want to be a good father is to want to make my kid the habit of being at home with the family. To keep a family tradition and make a citizen able to provide for the people.

Relatedly, fathers described wanting their child to grow up to be a good person and member of society, contributing to their communities, the world, and future generations (related to the cultural value of *colectivismo*). One Mexican-born father said, “I want her to be able to have a great heart so that she can help other people.” Further, an Ecuadorian-born father stated:

My greatest concern as a father is that they can be people for good, responsible and that they can contribute to being a better society or a better community, a better country, a better world for them and for the future generations that they have.

Finally, a Latino father born in Peru stated “I dream that she is a good person, a person with a good future who can contribute something to this life, to this world and that is my wish, that’s my motivation, to being a good father.” These men spoke to not only their generativity, but the future generativity of their children, and showcased the more community-oriented, collectivistic values of *bien educado* and *familismo.* Ultimately, for many fathers it was not just about their child turning out well, but that their child would be of service to others and make a lasting impact on their family, community, and world.

### Motivated by their Relationship with their Child

A clear source of Latino fathers’ perceived parenting motivations, especially among immigrants, was their regard for their relationship with their child, reflecting the value of *personalismo*. This theme included four dimensions: (a) wanting to have a positive father-child relationship, (b) loving their child, (c) wanting to watch their child grow up, and (d) wanting to be a good role model. Several fathers, mainly immigrants (75%), reported that they wanted to have and maintain a strong, positive relationship with their child. For example, a Latino father from El Salvador said “It is very important for me to be a friend for my daughter, that is something I want before being a father.” Fathers also mentioned wanting their child to have a secure base, or for their child to have a comfort zone in them. These men communicated wanting their child to feel loved and not alone, as well as to feel securely attached with the family.

Numerous fathers described that they felt motivated to be a “good” parent simply because they loved their child. A U.S.-born Latino father highlighted his strong feelings of love for his son, “The fact that he’s here and he’s the beacon in our life for both my wife and myself.” Another U.S.-born Latino father stated “First and foremost, my love for him. Ever since I met him, I suddenly fell in love with him, and so that makes me want to be a good parent.”

Incorporated within this dimension, many fathers also said that the child themself is what motivated them as fathers. Often, fathers would respond to the interview question simply by saying “she does,” or “him,” and pointing at their child. Further, a father born in Mexico said:

Her smile, at first when I first found out that I was going to be a parent I was scared, I had no idea what I was going to do, and now I am here and like, it just random thoughts pop up in my head of her make me want to work harder, she is the motivation herself.

Fathers’ desire to watch their child grow up was expressed as wanting to be present for their child’s life journey and to see their development unfold over time. One Mexican-born father said “What makes me want to be a good parent? To see how she’s growing every day.” Additionally, a father born in Bolivia said “Just being there for his experience, or being present for his growth, that’s it.” Fathers, many of whom were immigrant (over 3/4), also wanted their child to have someone to look up to and to provide a good example for their child’s future. They also wanted their child to think of them as having been a good role model once they are grown. A father born in El Salvador said:

Sometimes I believe that when my daughter grows up she will have the image of her dad who woke up at 5 in the morning to fight for her… Because right now my daughter even if you do not believe it, but she is retaining information like a computer a lot of images and a lot of things that she sees me doing right now and she just observes… and she is taking in all these images.

Another Latino father born in Ecuador described his son’s perceptive nature:

I believe the fact that knowing that he is growing up and that you are being an example, then everything he sees me do or the things he hears me say he’ll try to imitate them and if I do not do things well, he also won’t because I am a mirror for him. So, I think that the fact of, uh, knowing how I do things so that he can follow the example and, little by little realize that we are transmitting good things to him.

Both of these men noted how their children were observing and learning from their actions, which motivated them to be a good influence and model positive behavior.

### Intrinsic Motivations

Some fathers, especially immigrant fathers (almost 3/4), described wanting to be involved parents due to internally-driven reasons, such as it (a) coming naturally, (b) bringing them joy and satisfaction, (c) contributing to their personal development and growth, and (d) valuing their family. Some fathers said that being a good parent was just “natural” or came from within, arising from normal feelings that accompany being a father. Several men said being a “good” father was standard and expected, and indicated they had never even considered the question before. This was most commonly described by U.S.-born men (2/3). For instance, a U.S.-born father of Mexican descent said “I mean, why wouldn’t you want to be a good parent? I don’t know, I feel like, she’s just, you just want to make her happy, you know, your baby.”

Many fathers, most commonly immigrant fathers (over 3/4), reported that being a “good” parent brought them personal joy and satisfaction. These men felt a sense of great happiness and fulfillment from their role as a father. One man born in Venezuela said “I guess I can just enjoy it, I love to be a parent and really this is the most wonderful thing.” Some fathers, all of whom were immigrant, also mentioned how being a father contributed to their own personal growth and development. For instance, being a father helped some men to become more responsible or just to be a better person. A father born in Mexico said “…you know, keeps me motivated for going to work and doing my job right and getting more, trying to get more…”.

Some fathers (3/5 of whom were immigrant) reported that their personal value for their family, including their relationship with their partner, motivated them. They wanted to be involved parents because they loved and strongly valued their family, and also because of their spouse’s love for their child. One U.S.-born Mexican father reflected this internal value of *familismo*, “My wife considers me to be a good parent because, you know, our relationship has been pleasant to have, so I really want to reflect that to our son so…he may do the same in the future.” Another father who was born in Mexico said:

What makes me want to be a good father is to give a good example for my daughter, for my wife, to have a good communication with my family, want my child to have good values, teach my child to respect to my wife, wanting to be a good example for my daughter, and not wanting to be a bad example. Wanting to be the base of my family, so they respect me, that I respect them, and be good to them.

### Sense of Duty and Responsibility

Some fathers described wanting to be a “good” parent because they felt external pressures and perceived expectations to do so, perhaps related to aspects of *familismo*, *caballerismo*, and *respeto* that emphasize prioritizing family obligations, loyalty, and honor. Specifically, several fathers (most of whom were U.S.-born, 2/3) said that they felt being a “good” father was their duty or responsibility. For example, Latino fathers reported statements such as: “I feel like it’s my moral duty,” and “…I need to help them out.” Notably, while this extrinsically-oriented motivation emerged as a theme in the data, fewer fathers overall mentioned this influence compared to the other themes described above. We also note that there was great overlap in the themes within individual father’s responses, and men often reported a combination of several different motivating factors, such as wanting to instill good values, having a happy child, and feeling obligated to do their best.

## Discussion

Our thematic analysis revealed that the reasons for why first-time Latino fathers in the U.S. want to be positively engaged with their children are multiple. Among the most salient are their experiences with their own parents, their desire to rear well-adjusted children, the value they place in their relationship with their child, an intrinsic desire and personal benefits, and a sense of duty and responsibility. These perceived parenting motivations are theorized to underly fathers’ parenting behaviors and actions in being “good” parents – whatever that may look like and mean to them individually. We also found that perceived parenting motivations varied by fathers’ nativity status. Our findings provide additional context, meaning, and nuance to Cabrera and colleagues’ (2014) model of father-child relationships.

In talking about their sources of meaning and what motivates them to be “good” parents, Latino fathers made optimistic statements about reclaiming or remaking the past, making the most of their present, and building a strong future for their child. As a whole, fathers were not just focused on day-to-day factors but rather viewed parenting as a life-long process shaped by experiences that happened when they were young and outcomes that could happen when their children are grown adults. Fathering motivations rooted in their past centered around fathers’ personal rearing history and childhood experiences. Drawing upon past modeling experiences from one’s own parents appeared to be particularly relevant for immigrant fathers, whereas a lack of positive early experiences was more salient for U.S.-born fathers. In terms of the present, Latino fathers were motivated by their desire to have a well-adjusted child and a positive father-child relationship, and by various internal (especially among immigrant men) and external (especially among U.S.-born men) influences. Finally, regarding the future, fathers noted their desire to rear a well-adjusted child and to maintain their father-child relationship long-term. A focus on the quality of the father-child relationship appeared more frequently for immigrant fathers, whereas U.S.-born fathers were more likely to report focusing on their child’s future success.

### Rearing History

Findings from our study support Cabrera and colleagues’ (2014) heuristic model that fathers’ own rearing history is a predictor of their involvement with their children. This presented in several ways: fathers accepted good parenting, rejected poor parenting, and wanted to give their child what they never had, including a present and involved father. This is consistent with other studies that find that fathers’ own parenting experiences (especially with their fathers) informs current parenting behaviors (Cooper et al., 2019; Taylor & Behnke, 2005). The attachment quality men have with their parents has been shown to relate to their attachment relationship with their own children, sometimes positively aligned, and other times in compensatory ways (Volling & Belsky, 1992). This also reflects Taylor and Behnke’s (2005) qualitative findings of Latino fathers’ transmission versus transformation of behaviors they experienced from their own fathers. Further, low-income, ethnic-minority men’s relationships with their own fathers have been tied to the quality of interactions they have with their children, such that men who perceived their fathers as being highly accepting toward them were more responsive with their own infants (Shannon et al., 2005). Our findings add to this literature by showcasing the importance and complexity of intergenerational factors among Latino fathers.

### Desire to Rear a Well-Adjusted Child

Other theoretical influences of parenting motivation include children’s characteristics and developmental outcomes (Cabrera et al., 2014), which our findings also support. Fathers in this study were commonly driven to be “good” parents because of the type of child they wanted to rear – ones that would be happy, healthy, moral, successful, and good members of society, with sufficient resources and opportunities. These Latino fathers’ wishes for their children’s wellbeing echo the findings of ample research that has examined parents’ (mostly mothers’) developmental goals for their children (Suizzo, 2007; Tamis-LeMonda et al., 2008). Similarly, past studies have underscored the importance of intergenerational transmission of cultural values to children in Latine families (Aldoney & Cabrera, 2016; Taylor & Behnke, 2005). For instance, *educación*, *respeto*, *colectivismo*, *familismo*, and *caballerismo* (positive aspects of machismo) are known to be important values for parents to pass on (Arciniega et al., 2008; Cabrera & Bradley, 2012; Campos, 2008; Halgunseth, 2019). They include education and morality, placing others before oneself, showing respect, and treating others with dignity. Such current motivations for Latino fathers’ involvement with children (e.g., wanting their children to have good morals and to value the family) align well with these traditional cultural parenting goals, and support literature that demonstrates that most parents want their children to be well-adjusted people who embody a range of individualistic and collectivistic values (Aldoney & Cabrera, 2016; Campos, 2008; Suizzo, 2007).

### Father-Child Relationship

Consistent with Cabrera and colleagues’ (2014) framework and with the cultural value of *personalismo*, the quality of the parent-child relationship was an influential factor among our sample of Latino fathers. Although the bulk of research on parent-child relationship quality has focused on its unidirectional effects on children’s wellbeing, transactional models of development and family dynamics argue that parent-child relationships are dynamic and bidirectional, and thus mutually affect fathers as well (Cabrera et al., 2014; Schermerhorn & Cummings, 2008). The reciprocal nature of parent-child relationships was also evidenced by fathers in this study who said that being a parent contributed to their own personal growth and development, and it brought them feelings of joy and satisfaction. While some studies have tested reciprocal links between child adjustment and father involvement, no studies to our knowledge have examined the influence of father-child relationship quality on fathers’ parenting beliefs and cognitions. Our findings suggests that Latino fathers highly value maintaining this close relationship over the long-term and are motivated to serve as positive role models for their children as they grow up.

### Social Networks

Fathers in this study did not discuss their social networks or community as influencing their parenting beliefs, contrary to existing theory (Cabrera et al., 2014). Researchers have identified the benefits of social support networks for promoting fathers’ involvement with children (Castillo & Sarver, 2012), though this is primarily among non-resident fathers, which our sample did not include. Regardless, some literature has asserted that Latine adults are especially likely to rely on social support from family as compared to other racial-ethnic groups, and that social support is a particularly helpful resource for immigrant parents who have been separated from their extended family and face demographic risks (e.g., low-income and education; Taylor et al., 2015). One study found that Mexican-origin mothers reported significantly higher levels of social support than fathers, and mothers’ but not fathers’ social support was related to their own parenting behaviors (Taylor et al., 2015). This may be due to the fact that women are more likely than men to seek out and rely on social support in the first place. Indeed, past research has documented the challenges men often face in developing close friendships with other men because of adherence to stereotypical masculine norms (e.g., unemotional, homophobic, tough; Vierra et al., 2023) which may also overlap with aspects of *machismo* (Planalp et al., 2021).

### Mother-Father Relationship

Unlike past findings and theory, our participating fathers did not often mention their partner or co-parent as a perceived motivating influence. Ample work has identified the importance of mothers (e.g., mother-father romantic relationship, coparenting relationship, fathers’ perceptions of his partner’s support) in predicting fathers’ parenting beliefs, attitudes, and involvement (Bouchard et al., 2007; Ross-Plourde et al., 2022). However, our current findings challenge the notion of a strong perceived maternal influence – few Latino fathers mentioned their partner when describing their perceptions of what motivated them as parents. Rather, the fathers largely described aspects of themselves, their children, or their family more generally as primary motivators. Other studies have similarly noted a lack of maternal influence on fathers. For instance, a study of low-income, ethnic-minority fathers (63% resident) found that men’s relationship quality with their partners was unrelated to their father-infant interaction quality, which the authors state may have been because the vast majority of the fathers reported high-quality partner relationships (Shannon et al., 2005). It is possible that mothers’ influences on fathers’ parenting beliefs are less defining for co-habitating couples, and past work has identified mostly high-quality, cooperative co-parenting relationships among Latine couples in the U.S. (Cabrera et al., 2021).

### Differences by Nativity Status

Many of the differences in perceived parenting motivations between U.S.-born and immigrant fathers in this study can be tied to differences in cultural socialization and internalization of beliefs and values of their countries of origin. For instance, most of the men who modeled after their own parents, and those who rejected the modeling of their parents, were immigrant fathers. This speaks to the nuances of social learning through vicarious experiences with role models. Our findings also mirror those of Taylor and Behnke (2005), whereby the majority of their Mexican-national sample of fathers, but not their U.S. Latino sample, said they simultaneously replicate *and* reform the early experiences they had with their own fathers. It is possible that immigrant fathers’ beliefs and behaviors are especially influenced by their own parents because of their endorsement of cultural values such as *familismo* which emphasize family closeness, and the view that one’s identity is rooted in their family (Planalp et al., 2021). Alternatively, U.S.-born fathers made up the majority of those who reported wanting their child to “have more” than what they had growing up. Perhaps for the immigrant fathers, this was not as relevant because their circumstances in the U.S. were an improvement over their sending context (i.e., the conditions in their country of origin and factors for why they left; Taylor & Behnke, 2005), and thus they felt their children were already getting more than what they had themselves. Most of the fathers who reported wanting to serve as roles model for their children and who were motivated to have positive relationships with their children were immigrant fathers (reflecting *familismo and personalismo)*. Relatedly, most of the fathers who described wanting their children to have good morals and values were immigrants (connected to *respeto and educación)*, but over half of the fathers who reported wanting their child to have a successful future were U.S.-born. This difference may be due to U.S.-born fathers’ socialization experiences that promote Western-oriented traits like individualism and personal achievement. Finally, the large majority of men who were intrinsically motivated (e.g., fathering brought joy and satisfaction; they value their family) were immigrant fathers, which again reflects a strong sense of *familismo*. Conversely, most of the men who said being a good father was just “natural” were those born in the U.S., and this could be because in the U.S., it is now culturally and societally expected that fathers are equally involved in childrearing as are mothers.

### Limitations and Future Directions

Though this study makes contributions to the Latine parenting and fathering literatures, it also contains a number of limitations. Since these data were collected as part of a larger parenting intervention (BB2), a limited amount of home visiting time could be spent on parent qualitative interviews. As such, a main limitation was the highly-structured nature of the interviews and inability to probe parents further about their responses. This was sometimes an issue particularly for Spanish-speaking participants, who occasionally misinterpreted a question but who were then not always redirected by research assistants. Another limitation was the translation of the Spanish transcripts, based on the vernacular of 10 different Spanish-speaking countries of origin of the fathers, as well as several different Spanish vernaculars of research assistants. We often found subtle differences in the Spanish to English translations based on the research assistant’s form of Spanish and country of origin. Though our procedure involved three different native Spanish-speakers verifying each transcript, variations in interpretation and translation are possible. Relatedly, all coding was conducted with the English transcripts; the research team could not directly code from the Spanish transcriptions because none of the authors are fluent in Spanish. As such, we may have missed nuances in fathers’ meanings, though we aimed to mitigate this by discussing any questions with RAs who are fluent in Spanish. Finally, this sample was limited to first-time Latino fathers with 18-month old children who were voluntarily participating in a parenting intervention. This restricts the generalizability of the findings to other populations of U.S. Latino fathers, including fathers with multiple children or those at different stages in their parenting journeys. The exclusive focus on first-time fathers limits insights into how fathering motivations may evolve with subsequent children. Fathers with multiple children could exhibit differing motivations influenced by prior parenting experiences, learning from past successes or challenges, and altered family dynamics. Fathering beliefs and attitudes may also change as children age, with older children or adolescents presenting distinct developmental stages, needs, and relational dynamics compared to infants or toddlers.

Future work should continue to explore the within-group variability (e.g., by nativity status or acculturation status) of Latino fathers’ parenting beliefs, attitudes, and cognitions, given the potentially predictive influence of these cognitions on fathering behaviors. This work should also be conducted longitudinally to better understand how fathers’ motivations change over time as children get older, and as new children are born into the family. Such work could be used to understand the dynamic nature of parenting motivations, the connection between fathers’ motivations and their paternal involvement, and the relation from motivations to children’s developmental outcomes (as well as reciprocal influences). In addition, our participating fathers rarely mentioned their partner or anyone else in their social network as a primary motivation for their involvement. It may be worth further exploring these exclusions with other samples of fathers, as the sociocultural definition of masculinity and the role of the father in families continuously evolves over time. Intergenerational influences on Latino fathering should also be examined in future work. Although much past research on this topic has been directed at African American men, Latino fathers in this study reported nuanced and impactful relationships with their own parents, especially their fathers.

### Implications

Parenting motivations shape parenting behaviors, and parenting behaviors are known to shape children’s developmental outcomes (Cabrera et al., 2014). Accordingly, understanding perceived parenting motivations is important from an intervention standpoint, particularly for fathers who may be at risk of negative-quality or uninvolved parenting. These findings are also relevant for fathers who face social and structural barriers, and those who may endorse non-egalitarian gender roles, that can jeopardize their positive, continued involvement in caretaking and child rearing activities (Cabrera et al., 2008; Cabrera & Bradley, 2012; Coltrane et al., 2004). Focusing on parenting motivations offers a theoretically-grounded path of influence to fathers’ parenting behaviors; if service providers focus on behaviors alone without also observing the meaning alongside behaviors, interventions may be unsuccessful in creating lasting change in parenting. But, understanding a father’s perceived motivations and his sources of meaning can help researchers and service providers to more effectively promote (or discourage) certain parenting behaviors, if they can draw from what truly drives fathers to be “good” parents in the first place.

## Conclusion

This study is unique in its exploration of variability among an understudied and heterogeneous population in the parenting literature: U.S. Latino fathers. We employed a strengths-based approach in qualitatively exploring perceptions of parenting motivations among a relatively large sample of Latino fathers. Further, we contribute to the burgeoning literature on differences between immigrant and non-immigrant Latino parents living in the U.S. Men with greater motivation to parent well are likely to be more involved, remain involved, and provide higher quality parenting behaviors (Cabrera et al., 2014), which ultimately benefits their own wellbeing, their child’s wellbeing, and family functioning. We found that Latino fathers’ reasons for wanting to be a “good” parent align with findings from fathers (and mothers) across various backgrounds. Personal experiences with parents, love of children, and desire for the best for their children were common motives, and these motivations were linked to various time points from fathers’ past, present and ideal future. If researchers can better understand Latino fathers’ parenting motivations, as explained in their own words, we can better identify ways to support Latine families, positive paternal involvement, and children’s wellbeing.

## Data Availability

The study used primary data collection. Thus, the data are not available to the public.
